# Prognostic and clinical significance of syndecan-1 in colorectal cancer: a meta-analysis

**DOI:** 10.1186/s12876-015-0383-2

**Published:** 2015-10-30

**Authors:** Hao-tang Wei, Er-na Guo, Bao-guo Dong, Li-sheng Chen

**Affiliations:** Department of Colorectal & Anal Surgery, the First Affiliated Hospital of Guangxi Medical University, Shuangyong Road 6, Nanning, 530021 China; Institute of International Education, Guangxi Medical University, Nanning, 530021 China; Department of gastrointestinal surgery, the Third Affiliated Hospital of Guangxi Medical University, Nanning, 530031 China

## Abstract

**Background:**

Syndecan-1 plays a vital role in the suppression, transformation, and migration of several cancer types, including colorectal cancer (CRC). However, the prognostic and clinical significance of syndecan-1 in CRC remains conflicting. Therefore, we performed a meta-analysis to clarify this relationship.

**Methods:**

A comprehensive literature search for relevant studies published up to December 2014 was performed using PubMed, EMBASE, and Ovid library database. The odds ratio (OR) and pooled hazard ratio (HR) with their 95 % confidence intervals (CI) were used to estimate the effects.

**Results:**

Ten studies with 888 CRC patients were selected for evaluation. The results showed that syndecan-1 expression was lower in CRC tissue than in normal colorectal tissue (OR = 0.02, 95 % CI = 0.00–0.09), and lower in the advanced stage than in the early stage (OR = 2.24, 95 % CI = 1.14 − 4.42). Additionally, syndecan-1 expression was higher in well and moderately differentiated CRC than in poorly differentiated CRC (OR = 2.91, 95 % CI = 1.21–6.98); no significant difference was found in patients with or without lymph node metastasis (OR = 0.91, 95 % CI = 0.34–2.43) and distant metastasis (OR = 0.89, 95 % CI = 0.19-4.21). The pooled results showed that syndecan-1 expression was not associated with survival in CRC patients (HR = 0.93, 95 % CI = 0.86–1.01).

**Conclusion:**

This meta-analysis indicated that loss of syndecan-1 expression is associated with CRC development, histological differentiation, and clinical stage, but not with lymph node metastasis and distant metastasis. In addition, these findings fail to support the prognostic significance of syndecan-1 in CRC.

## Background

Colorectal cancer (CRC) is one of the most common gastrointestinal cancers. CRC patients with metastatic disease often have a poor prognosis [1]. Surgical resection is the main approach for the treatment of CRC but has limited effectiveness in patients with locally invasive or metastatic disease. Therefore, understanding the mechanisms of disease progression and identification of prognostic markers are essential in improving the treatment of CRC. Syndecan-1 (CD138), a transmembrane proteoglycan, plays a critical role in cell-cell and cell-extracellular matrix adhesion and acts as a growth factor coreceptor [2]. Syndecan-1 is highly expressed in normal epithelial cells, and loss of expression is associated with epithelial-mesenchymal transition and the transformed phenotype [3, 4]. Furthermore, many studies have shown that loss of syndecan-1 expression is associated with tumor development and progression [5–7].

Syndecan-1 has been reported to have prognostic value in many types of cancers such as hepatocellular cancer [8], laryngeal cancer [9], gastric cancer [10], and lung cancer [11]. Studies investigating the association of syndecan-1 expression with clinical parameters and prognosis in CRC patients have yielded inconsistent results. Fujiya et al. [12] found that CRC patients with syndecan-1 negative tumors had a high incidence of lymph node and liver metastasis. Furthermore, the survival rate in patients with syndecan-1 negative tumors decreased significantly in a stage-independent manner. Consistent with this, Wang et al. [13] showed that high preoperative syndecan-1 serum level was significantly associated with poor disease-free survival. However, Lundin et al. [14] showed that the cumulative 5-year survival was not significantly different between patients with strong and weak syndecan-1 expression. Hashimoto et al. [15] and Mitselou et al. [16] also failed to demonstrate the prognostic significance of syndecan-1 expression in CRC. Differences in sample size (i.e., statistical power) or other study parameters may have contributed to these discrepant findings. Meta-analysis is a quantitative method for combining data from multiple studies in a single study and provides more reliable results compared with individual studies. Therefore, we performed a meta-analysis to examine the relationship between syndecan-1 expression and clinical parameters and prognosis in CRC patients.

## Materials and methods

### Search strategy and selection criteria

The meta-analysis was performed in accordance with the guidelines of the Preferred Reporting Items for Systematic Review and Meta-analyses statement [17]. We systematically searched PubMed, EMBASE, Ovid library database, Chinese National Knowledge Infrastructure, Chinese Biomedical Database, and conference abstracts from recent presentations at international meetings, such as ASCO and ESMO, to identify suitable studies prior to December 2014. The Medical Subject Headings and text words used for the search were “colorectal neoplasms” or “colorectal cancer,” “syndecan” or “CD138,” and “prognosis.” The differences in truncation symbols and wildcards between databases were used for the retrieval strategy in order to identify potentially relevant studies. The searches were limited to human studies. Articles were also identified using the related articles function in PubMed. References within the identified articles were also searched manually. Potentially relevant articles were screened by two independent reviewers. Disagreements between the reviewers were resolved by discussion or upon consensus from a third reviewer. The study protocol was approved by the ethics committee of the First Affiliated Hospital of Guangxi Medical University.

### Inclusion and exclusion criteria

The inclusion criteria were as follows: 1) patients with confirmed diagnosis of CRC; 2) assessment of the association of syndecan-1 expression with clinical parameters and prognosis in CRC; and 3) detection of syndecan-1 expression in CRC tissue by immunohistochemistry (IHC). Exclusion criteria were as follows: 1) review articles, case reports, and animal studies; 2) cell line and human xenograft studies; 3) studies using methods other than IHC for detection of syndecan-1 expression; and 4) low-quality studies, including studies without data on patients’ demographic characteristics or IHC findings. When the same patient population was reported in several publications, only the most complete study was included in the analysis. Two reviewers independently assessed study eligibility. Disagreements between reviewers were resolved by consensus.

### Data extraction and quality assessment

Study data were assessed and extracted independently by two reviewers. Disagreements between the reviewers were resolved by discussion and consensus. Extracted data included author names, year of publication, sample source, number of male and female patients, tumor classification system, IHC methodology (antibody source, dilution), and clinical parameters (tumor stage, lymph node metastasis, distant metastasis). In the present study, in order to reduce the bias, we combined both the Dukes and TNM staging systems in the AJCC staging system (seventh edition, 2010) and reassessed the association between syndecan-1 expression and CRC.

The methodological quality of each study was assessed and scored according to the Newcastle-Ottawa Quality Assessment Scale [18]. The following items were assessed in each study: exposed cohort, ascertainment of exposure, outcome of interest, comparability of cohorts, assessment of outcome, and adequacy of follow-up. Studies with a score of 6 or higher were considered to be of high quality. Two reviewers independently assessed study quality. Quality scores were obtained by consensus.

### Statistical analysis

The pooled odds ratio (OR) with 95 % confidence interval (CI) was used to quantitatively determine the association between syndecan-1 expression and clinical parameters in CRC patients, whereas the hazard ratio (HR) with 95 % CI was used to quantitatively evaluate the association between syndecan-1 expression and patient survival. Heterogeneity among studies was evaluated using Cochran’s Q test and the I^2^ statistic. The pooled OR and HR were calculated using a fixed-effects model (Mantel-Haenszel method) in the absence of substantial study heterogeneity (I^2^ < 50 %). Otherwise, a random-effects model (DerSimonian-Laird method) was used. Sensitivity analysis was performed by removing studies on chemotherapy and removing each study in turn to test the reliability of the overall pooled results. Publication bias was assessed using Egger and Begger's tests. All statistical analyses were performed using Stata 11.2 software (Stata Corp, College Station, TX). A two-tailed *P* value <0.05 was considered statistically significant.

## Results

### Identification of relevant studies

**Fig. 1:**
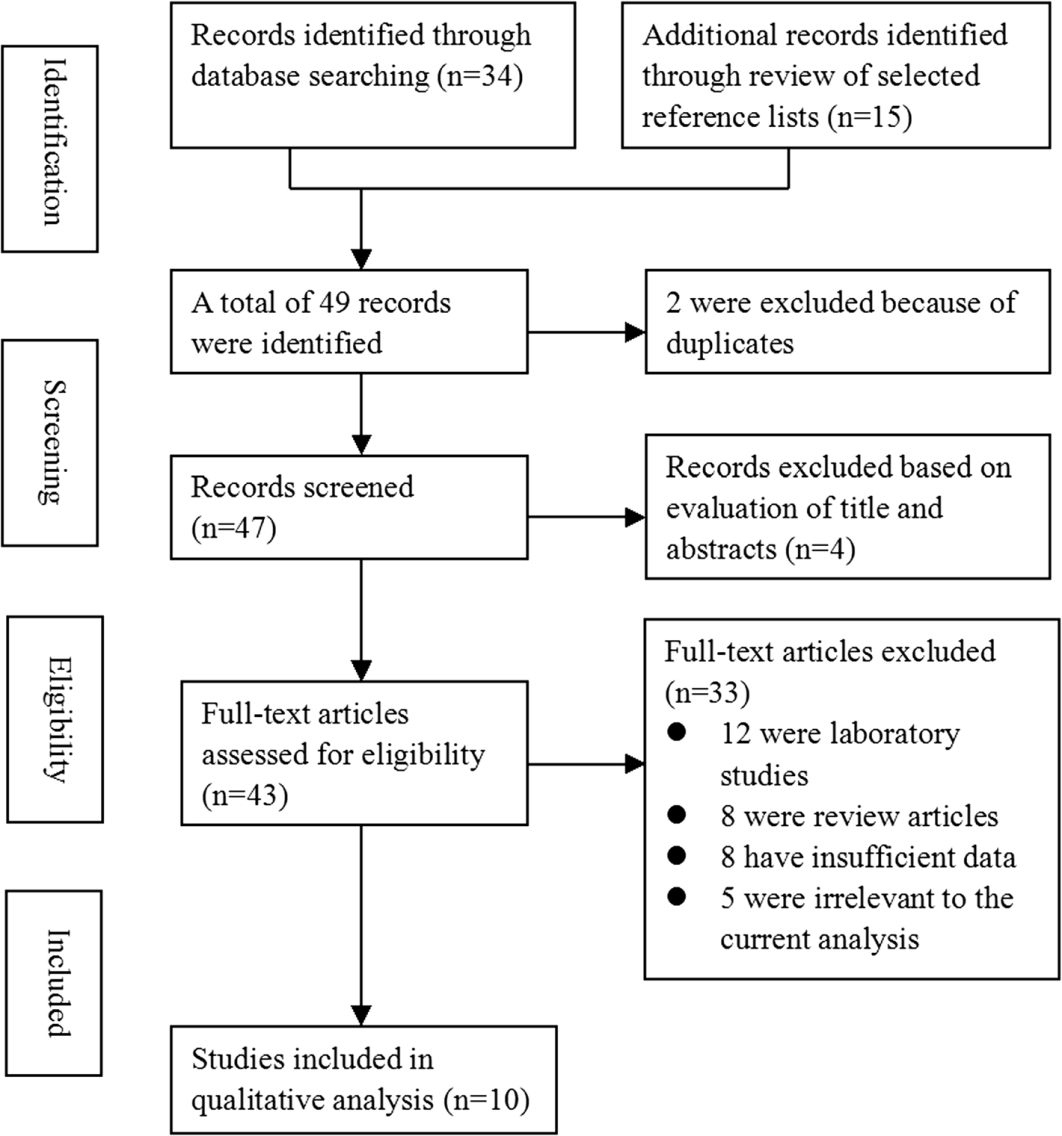
Flowchart of study selection

The initial literature search retrieved 47 potentially eligible studies based on the predefined selection criteria. After a detailed evaluation, 37 studies were excluded, including 12 laboratory studies, 8 reviews, 8 studies with insufficient data, and 9 irrelevant studies. Thus, 10 studies [12–15, 19–24] involving 888 patients were included in the meta-analysis (Fig. 1).

### Study characteristics and quality assessment

All included studies used IHC to detect syndecan-1 expression. Four studies [12, 14, 15, 21] provided follow-up data. Four [12, 13, 15, 19] and 6 [14, 20–24] studies used the TNM and Dukes classifications for tumor staging, respectively. The quality of the studies was high, with scores ranging from 7 to 9. Patient demographic data from each of the included studies are shown in Tables 1 and 2.

**Table 1:** Characteristic of selected studies in the meta-analysis

Author	Year/country	Gender	Tumor stage	Antibody	Dilution	Follow up	Quality
		(M/F)		source			Score
Wang et al	2014/China	25/27	TNM	Maixin	NA	40 mo	8
Yang et al	2010/China	33/25	Duke	Santa Cruz	1:50	5 yrs	8
Pap et al	2009/Romania	71/46	Duke	Fremont	1:50	NA	8
Hashimoto et al	2008/USA	79/52	TNM	Serotec	1: 100	38 mo	9
Qi et al	2008/China	33/19	Duke	Maixin	NA	NA	7
Feng et al	2007/China	20/11	TNM	Zhongshan	1:75	NA	8
Lundin et al	2005/Finland	118/119	Duke	Serotec	1:100	116 mo	9
Zhou et al	2005/China	33/19	Duke	Zhongshan	NA	NA	7
Zuo et al	2004/China	29/22	Duke	Zhongshan	NA	NA	7
Fujiya et al	2001/Japan	65/40	TNM	Santa Cruz	1:50	10 yrs	9

**Table 2:** Characteristic of selected studies in the meta-analysis

Author	Clinical parameters assessment (number)	Scoring system (Positivity cells)	Number of patients	Epitope retrieval method
Wang et al	T, L, H, D, F. (52)	histoscore = area × intensity^a^	High level (26), Low level (26)	NA
Yang et al	F. (58)	histoscore = area × intensity^a^	Positive (19), Negative (39)	HIER
Pap et al	T, L, H, D. (117)	Frequency of cells + intensity^b^	G0 (2), G1-3 (115)	pressurized steam cooking
Hashimoto et al	T, L, H, D, F. (131)	0 (< 5 %); 1+ (5 % – 25 %); 2+ (25 % – 75 %), 3+ (> 75 %)	0 (38), 1+(27), 2+(49), 3+(17)	antigen retrieval solution
Qi et al	T, L, H, D, E. (52)	0 (< 5 %); 1+ (5 % – 25 %); 2+ (25 % – 75 %), 3+ (> 75 %)	Positive (34), Negative (18)	HIER
Feng et al	T, L, H, D, E. (31)	0 (< 5 %); 1+ (5 % – 25 %); 2+ (25 % – 75 %), 3+ (> 75 %)	Positive (25), Negative (6)	HIER
Lundin et al	T, H, F. (237)	Weak: < 30 %, Strong: > 30 %	Weak (86), Strong (151)	NA
Zhou et al	T, L, H. (52)	0 (< 5 %); 1+ (5 % – 25 %); 2+ (25 % – 75 %), 3+ (> 75 %)	0 (18), 1+(13), 2+(14), 3+(7)	HIER
Zuo et al	T, L, H, D, E. (51)	0 (< 5 %); 1+ (5 % – 50 %); 2+ (> 50 %)	0 (31), 1+(17), 2+ (3)	HIER
Fujiya et al	T, L, F. (105)	– (< 5 %); + (5 % – 25 %); ++ (25 % – 75 %), +++ (> 75 %)	– & ± (36), + & ++ (69)	HIER

*NA* not available, *T* depth of infiltration, *L* lymph node metastasis, *D* distant metastasis, *H* Histological grade, *F* follow-up, *E* expression of tumor, *HIER* heat induced epitope retrieval

^a^The intensity of membranous staining was graded as negative (-), weak (+), moderate (++) and intense (+++). The percentage of cells was graded as follows: 0 (<5 %), 1 (5–25 %), 2 (26–50 %), 3 (51–75 %) and 4 (> 75 %). Imunohistochemical score (histoscore) = values of the intensity × the percentage of cells (area).^b^Frequency (F) of immunopositive tumor cells: F0: 0–5 %, F1: 6–30 %, F2: 31–75 %, F3: 76– 100 %; and the signal intensity (I): absent (0), weak (1), moderate (2), strong (3). Immunohistochemical grading (IG) system: IG0 (absent): F+I= 0–1; IG1 (weak): F+I=2–3; IG2 (moderate): F+I= 4; IG3 (strong): F+I= 5–6

### Syndecan-1 expression in CRC tissue

**Fig. 2:**
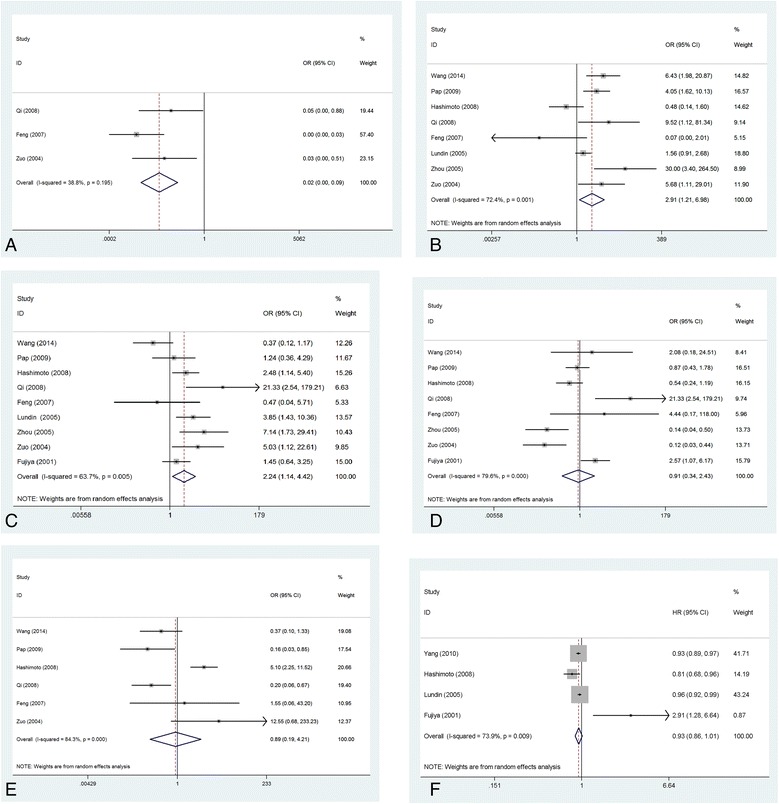
Meta-analysis of syndecan-1 expression in CRC. **a** CRC and normal colorectal tissue; **b** histological differentiation grade; **c** clinical stag; **d** lymphoid node metastasis; **e** distant metastasis; **f** survival time. OR: odds ratio; HR: hazard ratio. The pooled OR and HR were calculated using a random-effects model

In our study, we classified syndecan-1 expression into two types according to the IHC results: negative/low syndecan-1 expression or positive/high syndecan-1 expression. Three studies involving 194 samples assessed syndecan-1 expression in CRC and normal colorectal tissues. Significant heterogeneity across the studies was not observed (I^2^ = 38.8 %, *P* = 0.195; Fig. 2a). Syndecan-1 expression was lower in CRC tissues compared with normal colorectal tissues (fixed-effects model: OR = 0.02, 95 % CI = 0.00 − 0.09).

### Association of syndecan-1 expression with clinical parameters in CRC

The association of syndecan-1 with clinical parameters is shown in Figs. 2b-e. Syndecan-1 expression was higher in well and moderately differentiated CRCs compared with poorly differentiated CRCs (random-effects model: OR = 2.91, 95 % CI = 1.21 − 6.98). However, significant heterogeneity across the studies was observed (I^2^ = 72.4 %, *P* = 0.001). After combining both the Dukes and TNM staging systems in the AJCC staging system, we found that syndecan-1 expression was significantly lower in advanced-stage (T3-4 stage) than in early-stage (T1-2 stage) CRC (random-effects model: OR = 2.24, 95 % CI = 1.14 − 4.42; I^2^ = 63.7 %, *P* = 0.005). Syndecan-1 expression was not significantly different between patients with and without lymph node metastasis (random-effects model: OR = 0.91, 95 % CI = 0.34 − 2.43) or distant metastasis (random-effects model: OR = 0.89, 95 % CI = 0.19 − 4.21). However, significant heterogeneity across the studies was observed (lymph node metastasis: I^2^ = 72.4 %, *P* = 0.001; distant metastasis: I^2^ = 84.3 %, *P* < 0.000).

### Association of syndecan-1 expression with prognosis in CRC

Four studies provided data regarding the association of syndecan-1 expression with overall survival of CRC patients. However, we excluded one study (Wang et al. [13]) that provided data on disease-specific survival time from the pooled analysis. Heterogeneity among the four studies was significant (I^2^ = 73.9 %, *P* = 0.009). The pooled results showed that syndecan-1 expression was not associated with prognosis in CRC patients (random-effects model: HR = 0.93, 95 % CI = 0.86 − 1.01; Fig. 2f).

### Sensitivity analysis and publication bias

The patients in the study by Wang et al. [13] study received chemotherapy, hence to eliminate the effect of chemotherapy and test the robustness of the overall results, we excluded data of Wang et al.’s study from the sensitivity analysis and found that the results were in line with the overall results (data not shown).

**Fig. 3:**
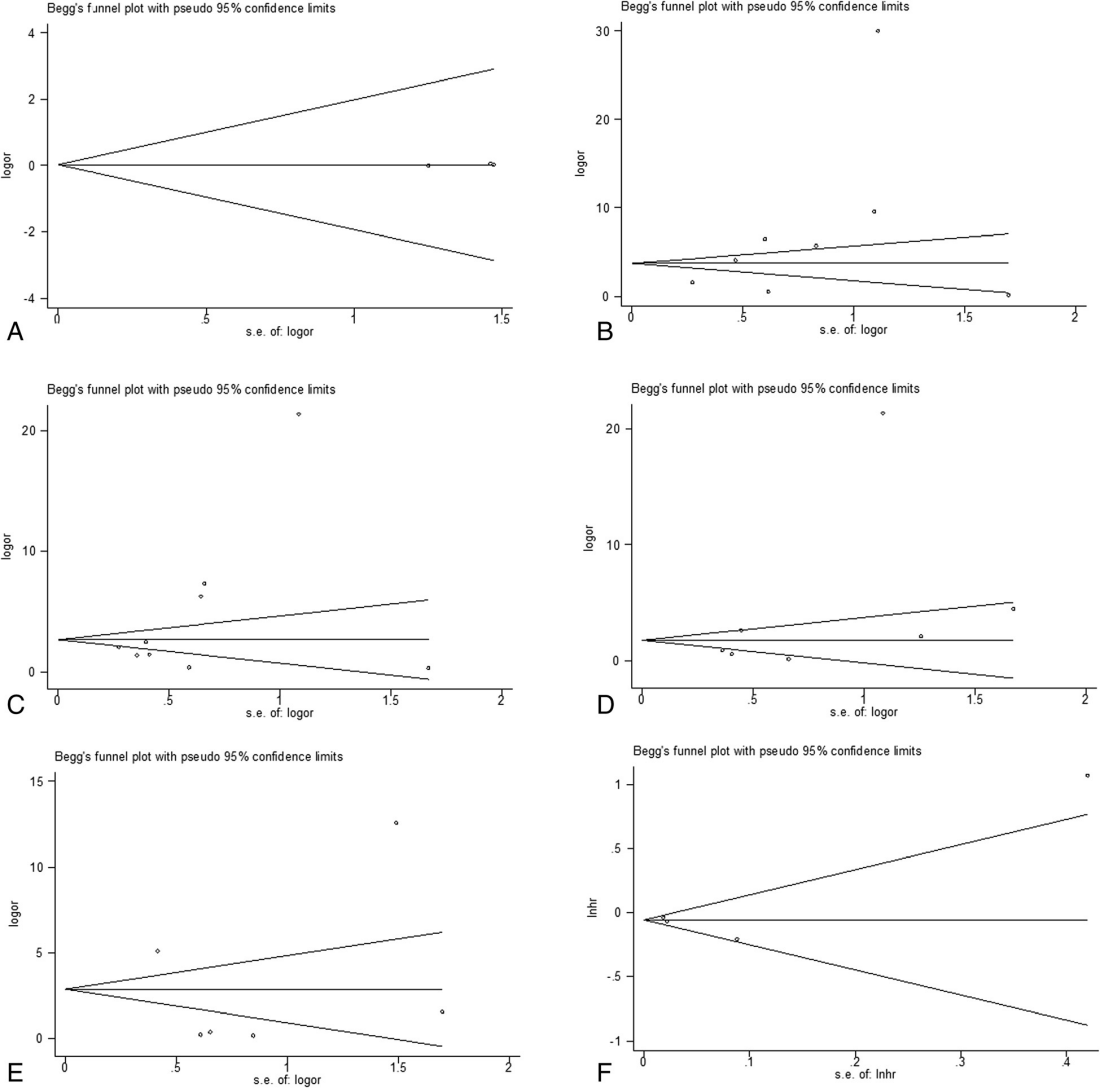
Funnel plots suggesting there were no publication biases in the meta-analysis. **a** CRC and normal colorectal tissue; **b** histological differentiation grade; **c** clinical stag; **d** lymphoid node metastasis; **e** distant metastasis; **f** survival time

We also performed sensitivity analysis by removing each study in turn from the analysis. The pooled ORs and HRs were not significantly changed, indicating the stability of our analyses. The funnel plots were largely symmetrical, and the results of the Egger and Begger's tests showed no evidence of significant publication bias. See Fig.3.

## Discussion and conclusion

Syndecan-1 is a member of a four-member family of cell surface heparan sulfate proteoglycans. Syndecan-1 is known to play important roles in the regulation of epithelial homeostasis, proliferation, and migration [25]. Syndecan-1 has also been shown to suppress transformation and migration in several cancer cell lines. Syndecan-1 expression is often lost in a wide range of cancer cell lines and cancer tissues. Several mechanisms have been suggested to explain the relationship between loss of epithelial syndecan-1 expression and cancer progression. Cancer development and progression is accompanied by the disruption of cell-cell and cell-matrix adhesion [26]. As an adhesion molecule, syndecan-1 influences the tumorigenic activity of cancer cells by altering extracellular matrix adhesion and cell morphology [27]. Disruption of cell-cell and cell-extracellular matrix adhesion by epithelial syndecan-1 loss leads to cancer cell growth, migration, and dissemination and thus, to poor patient prognosis [28, 29].

In the present meta-analysis, we found that syndecan-1 expression is lower in CRC tissues compared with normal colorectal tissues. This finding indicates that loss of syndecan-1 expression in colonic epithelial cells is associated with CRC development. The pooled data also showed that syndecan-1 expression is higher in well and moderately differentiated CRCs compared with poorly differentiated CRCs. Thus, syndecan-1 expression was associated with histological grade in CRC. In addition, the pooled results suggested that syndecan-1 expression is lower in the advanced stage than in the early stage. However, our analysis failed to demonstrate an association of syndecan-1 expression with lymph node metastasis. This finding is inconsistent with that reported by Pap et al. [24], but agreed with that reported by Fujiya et al. [12]. The reasons for these contradictory findings are not known. Thus, further investigation is necessary to clarify this association.

The association of syndecan-1 with clinical characteristics differs according to cancer type. Syndecan-1 has been associated with a high incidence of metastasis in patients with poorly differentiated hepatocellular carcinoma [8]. However, syndecan-1 expression was not different between early and advanced-stage pancreatic cancer [30]. Although reduced *syndecan-1* mRNA expression conferred a growth advantage to cervical carcinoma cells [31], syndecan-1 expression was not associated with clinical stage or outcome in cervical carcinoma patients [32]. Currently, the AJCC seventh edition TNM classification has become the principal method for prognosis assessment in CRC. In our study, after combining both the Dukes and TNM staging systems in the AJCC TNM staging system and reassessing the association, we found an association of syndecan-1 expression with clinical stage, suggesting that syndecan-1 expression reduced in the advanced stage of CRC. Regarding the prognostic value of syndecan-1, our analysis failed to show an association between loss of syndecan-1 expression and survival time in CRC patients. However, although the four studies included had 531 patients, which seems to provide sufficient statistical power, the pooled HR is approximately 1 for three out of the four investigated studies, and there was significant heterogeneity across the studies, indicating that the prognostic value will be limited and further study focusing on this parameter is needed.

Our meta-analysis is the first to clarify the relationship between syndecan-1 expression and clinical parameters and prognosis in CRC. Our meta-analysis was able to overcome the small sample size of individual studies, thereby enhancing statistical power and providing a more reliable estimation of these associations. In addition, the results indicated the absence of significant publication bias, suggesting the robustness of the results. To date, IHC is the most common method used to detect the expression of cytokines at the protein level, while other methods, such as PCR, that detect expression at the gene level have also been used widely. These methods are mutually complementary. In our study, we chose IHC because most studies used this method to detect the expression of syndecan-1, and hence we could obtain a large sample size, which would lead to more reliable pooled results. However, further meta-analysis using other methods is warranted to confirm our results.

Our study has several limitations and thus, the results should be interpreted cautiously. First, significant study heterogeneity was present for several parameters, which decreased the robustness of the conclusions. Random-effect models and sensitivity analysis were used to reduce the impact of study heterogeneity on the meta-analysis results. Second, although 10 studies were included in the analysis, the number of patients was relatively small. A larger sample size is needed to obtain more reliable results. Third, the possibility of information and selection biases and unidentified confounders could not be completely excluded, as all included studies were observational. Fourth, although we searched several databases, other databases, such as Oncomine database, were not searched. Therefore, some relevant studies may have been missed, which may have undermined the robustness of the results. Fifth, CRC incidence greatly differs between Asian and Caucasian countries, but most patients included in our study were of Asian ethnicity, which may cause some selection bias. Finally, not much consensus exists on the evaluation of immunohistochemistry data. Thus, the pooled data in present study are subject to the bias caused by the various scoring systems used. Therefore, our results should be interpreted with caution.

In conclusion, the results of our study demonstrate that loss of syndecan-1 expression correlates with histological grade and tumor stage, but not with lymph node metastasis or distant metastasis, in CRC. Furthermore, syndecan-1 expression does not have prognostic value in CRC patients.

## Abbreviations

CRC: Colorectal cancer
OR: Odds ratio
HR: Pooled hazard ratio
CI: Confidence interval
IHC: Immunohistochemistry
